# Editorial: Early Detection and Early Intervention Strategies for Cerebral Palsy in Low and High Resource Settings

**DOI:** 10.3390/brainsci12080960

**Published:** 2022-07-22

**Authors:** Atul Malhotra

**Affiliations:** Department of Paediatrics, Monash University, Melbourne, VIC 3168, Australia; atul.malhotra@monash.edu

Cerebral palsy (CP) is the most common physical disability in childhood. Early detection and early intervention for cerebral palsy is now well-proven, evidence-backed, and slowly gathering momentum in hospitals and clinics around the world [1]. In this special developmental science issue of *Brain Sciences*, eight topical papers on the topic are presented. The focus of this Special Issue was on CP diagnosis and intervention (especially early detection and treatment) in both low- and high-resource settings.

Two papers feature work on the early detection of CP from high-resource clinical settings in Australia. Velde et al. described the single-centre implementation of early diagnosis in a CP clinic in NSW, Australia [2]. They showed that by using assessments based on current recommendations for diagnosis, CP can be reliably diagnosed well before 12 months of age. Furthermore, the clinic model is acceptable to parents and referrers, and supports access to CP-specific early-intervention therapies. In another study, Connors et al. described the utility of early neonatal assessments in the early diagnosis of CP in a well-established early diagnosis clinic in Victoria, Australia [3]. They showed that neonatal writhing general movement assessments were not significantly associated with early CP diagnosis in extremely preterm infants, but the Hammersmith neonatal neurological examination (HNNE) did correlate significantly with fidgety general movements and Hammersmith infant examination (HINE) at 3–4 months of age. Neonatal assessments (writhing general movements, HNNE) may thus serve as useful tools to stratify high-risk infants early in the infant period.

Five papers in this Special Issue focused on CP diagnosis and management in low- and middle-income settings. King et al. reviewed the literature on early diagnoses of CP in LMICs, highlighting the paucity of evidence and published studies from LMICs in this field [4]. Mushta et al. reviewed the epidemiology of CP in children and adolescents in Arabic-speaking countries [5]. Again, substantial knowledge gaps in the epidemiology of CP were identified in these settings, and the authors urged clinicians and authorities to follow successful examples of well-established CP registries from other jurisdictions. The next three studies explored service utilisation, community interventions, and a social business model of early intervention and rehabilitation for people with disability in LMICs. A case study of a rural early-intervention and rehabilitation centre in Bangladesh was presented by Al Imam and colleagues [6]. It highlighted the sustainability of such a model centre in these settings, which is critical for the long term success of such initiatives. Similarly, Karim et al. showcased a quasi-experimental study of an early-intervention community centre in Bangladesh, with promising outcomes [7]. Finally, Al Imam et al. assessed the predictors of rehabilitation service utilisation in the LMIC setting [8]. They identified that the child’s age, functional status and associated impairments, parental education and economic status influenced the rehabilitation utilisation among children in LMICs. These findings could help policymakers and service providers to improve and increase access to rehabilitation, and improve the equity to care.

In summary, the early diagnosis and early intervention of CP is critical for improving the long-term functional outcomes of CP children and their families. This Special Issue showcases some of the work which is being carried out in this field. Most importantly, it further highlights the work needed in LMIC settings to improve understanding of epidemiology of CP, and access to early diagnosis and intervention for these communities.
